# 2-(Cyclo­hexa-1,4-dien­yl)-2-(4-methoxy­phen­yl)-*N*,*N*-dimethyl­ethanaminium chloride

**DOI:** 10.1107/S1600536809027627

**Published:** 2009-07-18

**Authors:** Wei-Jian Lou, Xiu-Rong Hu

**Affiliations:** a Department of Pharmacy, Sir Run Shaw Institute of Clinical Medicine, Zhejiang University, Hangzhou, Zhejiang 310016, People’s Republic of China; b Center for Analysis and Measurement, Zhejiang University, Hangzhou, Zhejiang 310028, People’s Republic of China

## Abstract

In the title compound, C_17_H_24_NO^+^·Cl^−^, the cyclo­hexa-1,4-diene ring, which is almost planar, with a maximum deviation of 0.024 (4) Å from the mean plane, makes a dihedral angle of 66.4 (1)° with the benzene ring. In the crystal, inter­molecular N—H⋯Cl and C—H⋯Cl hydrogen bonds link the mol­ecules into an infinite chain along the *b* axis.

## Related literature

The title compound is an impurity  that is sometimes yielded during the preparation of venlafaxine, one of a novel group of anti­depressants characterized by their ability to  selectively inhibit the pro-synaptic re-uptake of both serotonin and noradrenaline, see: Vega *et al.* (2000 ▶); Yardley *et al.* (1990 ▶).
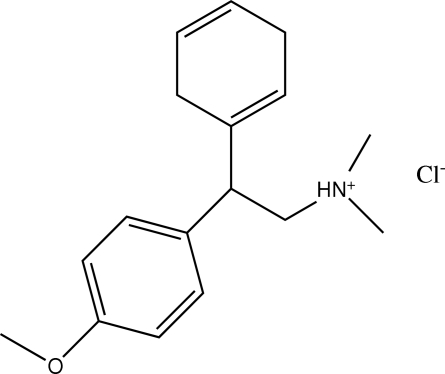


## Experimental

### 

#### Crystal data

C_17_H_24_NO^+^·Cl^−^
*M* *_r_* = 293.84
Orthorhombic, 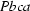

*a* = 11.1245 (7) Å
*b* = 10.9248 (6) Å
*c* = 28.9651 (16) Å
*V* = 3520.2 (4) Å^3^
*Z* = 8
Mo *K*α radiation
μ = 0.21 mm^−1^
*T* = 296 K
0.32 × 0.28 × 0.10 mm

#### Data collection

Rigaku R-AXIS RAPID diffractometer
Absorption correction: multi-scan (**ABSCOR**; Higashi, 1995 ▶) *T*_min_ = 0.927, *T*_max_ = 0.979
32170 measured reflections
4010 independent reflections
2102 reflections with *F* ^2^ > 2σ(*F* ^2^)
*R*_int_ = 0.081

#### Refinement

*R*[*F* ^2^ > 2σ(*F* ^2^)] = 0.060
*wR*(*F* ^2^) = 0.140
*S* = 1.00
4010 reflections
182 parameters
H-atom parameters constrained
Δρ_max_ = 0.61 e Å^−3^
Δρ_min_ = −0.46 e Å^−3^

### 

Data collection: *PROCESS-AUTO* (Rigaku, 1998 ▶); cell refinement: *PROCESS-AUTO*; data reduction: *CrystalStructure* (Rigaku/MSC, 2004 ▶), and Larson (1970 ▶); program(s) used to solve structure: *SIR97* (Altomare *et al.*, 1999 ▶); program(s) used to refine structure: *CRYSTALS* (Betteridge *et al.*, 2003 ▶); molecular graphics: *ORTEP-3 for Windows* (Farrugia, 1997 ▶); software used to prepare material for publication: *CrystalStructure*.

## Supplementary Material

Crystal structure: contains datablocks global, I. DOI: 10.1107/S1600536809027627/is2435sup1.cif

Structure factors: contains datablocks I. DOI: 10.1107/S1600536809027627/is2435Isup2.hkl

Additional supplementary materials:  crystallographic information; 3D view; checkCIF report

## Figures and Tables

**Table 1:** Hydrogen-bond geometry (Å, °)

*D*—H⋯*A*	*D*—H	H⋯*A*	*D*⋯*A*	*D*—H⋯*A*
N1—H101⋯Cl1	0.86	2.28	3.033 (2)	146
C17—H173⋯Cl1^i^	0.96	2.75	3.641 (3)	155

Symmetry code: (i) 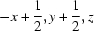.

**Fig. 1.**
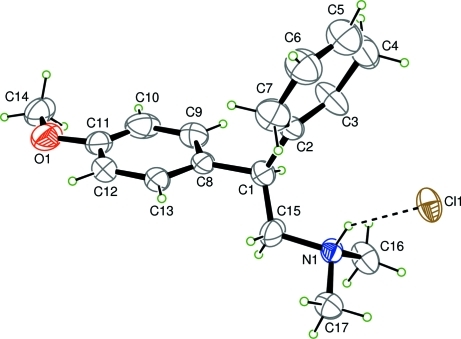
A view of (1). Displacement ellipsoids are drawn at 40% probability level and H atoms are shown as small circles of arbitrary radii.

**Fig. 2.**
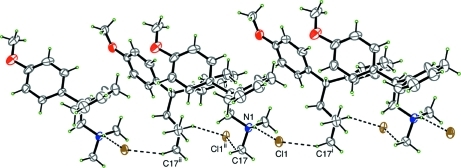
A chain of molecules in (1). Displacement ellipsoids are drawn at the 30% probability level and molecular interactions are shown in dashed lines [symmetry code: (i) 1/2 - x, 1/2 + y, z); (ii) 1/2 - x, -1/2 + y,z].

## supplementary crystallographic information

### Comment

The title compound is impurity of venlafaxine, which is a presentative of a novel group of antidepressants and is characterized by its ability to inhibit selectively the presynaptic reuptake of both serotonin and noradrenaline (Vega *et al.*, 2009; Yardley *et al.*, 1990). The asymmetric unit of the title compound contains a C_17_H_24_NO^+^ cation and a Cl^-^ anion. The ethylammonium N atom, N1, shows quatarnary character due to proton transfer from HCl and consequently bears the positive charge in the molecular cation. The N1 bond angles range from 108 to 115°, confirming the tetrahedral bond configuration, which is similar to that of venlafaxine hydrochloride (Vega *et al.*, 2009). An intermolecular hydrogen bond N1—H101···Cl1 is formed between atom Cl and atom N of ethylammonium. This hydrogen bond and a weak intermolecular interaction C17—H173···Cl1^i^ [symmetry code: (i) 1/2 - *x*, 1/2 + *y*, *z*)] link the molecules into a chain along the *b* axis (Fig. 2). Crystal packing is stabilized by these hydrogen-bond interactions and intermolecular interactions. In the crystal structure of the title compound, the cyclohexa-1,4-diene ring C2–C7 is almost planar, with a maximum deviation of 0.024 (4) Å at C4, which is revealed by torsion angle C2—C3—C4—C5 of -3.6 (6)° and C5—C6—C7—C2 of 0.5 (8)°. This cyclohexadiene plane forms dihedral angle of 66.4 (1)° with the benzene C8–C13 ring plane.

### Experimental

The crude product is supplied by Zhejiang Menovo Pharmaceutical Co., LTD. It was recrystallized from an ethanol solution, giving colorless crystals of (1) suitable for X-ray diffraction.

### Refinement

All H atoms were placed in calculated positions with C—H = 0.93–0.98 Å and N—H = 0.86 Å and included in the refinement in riding model, with *U*_iso_(H) = 1.2*U*_eq_(carrier atom).

### Crystal data

**Table:**

C_17_H_24_NO^+^·Cl^−^	*F*(000) = 1264.00
*M**_r_* = 293.84	*D*_x_ = 1.109 Mg m^−^^3^
Orthorhombic, *P**b**c**a*	Mo *K*α radiation, λ = 0.71075 Å
Hall symbol: -P 2ac 2ab	Cell parameters from 14141 reflections
*a* = 11.1245 (7) Å	θ = 3.4–27.4°
*b* = 10.9248 (6) Å	µ = 0.21 mm^−^^1^
*c* = 28.9651 (16) Å	*T* = 296 K
*V* = 3520.2 (4) Å^3^	Chunk, colorless
*Z* = 8	0.32 × 0.28 × 0.10 mm

### Data collection

**Table:**

Rigaku R-AXIS RAPID diffractometer	2102 reflections with *F*^2^ > 2σ(*F*^2^)
Detector resolution: 10.00 pixels mm^-1^	*R*_int_ = 0.081
ω scans	θ_max_ = 27.4°
Absorption correction: multi-scan (*ABSCOR*; Higashi, 1995)	*h* = −14→14
*T*_min_ = 0.927, *T*_max_ = 0.979	*k* = −14→14
32170 measured reflections	*l* = −37→37
4010 independent reflections	

### Refinement

**Table:**

Refinement on *F*^2^	*w* = 1/[0.0003*F*_o_^2^ + 1.2σ(*F*_o_^2^)]/(4*F*_o_^2^)
*R*[*F*^2^ > 2σ(*F*^2^)] = 0.060	(Δ/σ)_max_ < 0.001
*wR*(*F*^2^) = 0.140	Δρ_max_ = 0.61 e Å^−^^3^
*S* = 1.00	Δρ_min_ = −0.46 e Å^−^^3^
4010 reflections	Extinction correction: Larson (1970)
182 parameters	Extinction coefficient: 41 (8)
H-atom parameters constrained	

### Special details

**Table:**

Geometry. ENTER SPECIAL DETAILS OF THE MOLECULAR GEOMETRY
Refinement. Refinement was performed using all reflections. The weighted *R*-factor (*wR*) and goodness of fit (*S*) are based on *F*^2^. *R*-factor (gt) are based on *F*. The threshold expression of *F*^2^ > 2.0 σ(*F*^2^) is used only for calculating *R*-factor (gt).

### Fractional atomic coordinates and isotropic or equivalent isotropic displacement parameters (Å^2^)

**Table:**

	*x*	*y*	*z*	*U*_iso_*/*U*_eq_	
Cl1	0.42790 (7)	0.47986 (7)	0.74727 (3)	0.0737 (2)	
O1	0.4316 (3)	1.1886 (2)	0.52350 (11)	0.1084 (15)	
N1	0.3030 (2)	0.7155 (2)	0.71965 (8)	0.0560 (8)	
C1	0.3672 (4)	0.7774 (3)	0.63900 (12)	0.0798 (15)	
C2	0.4533 (3)	0.6719 (3)	0.63249 (10)	0.0648 (12)	
C3	0.4132 (3)	0.5682 (3)	0.61543 (14)	0.1020 (16)	
C4	0.4901 (5)	0.4576 (3)	0.60790 (19)	0.107 (2)	
C5	0.6194 (5)	0.4795 (5)	0.6190 (2)	0.116 (3)	
C6	0.6589 (4)	0.5804 (5)	0.6361 (2)	0.113 (2)	
C7	0.5830 (3)	0.6861 (3)	0.64451 (12)	0.0966 (16)	
C8	0.3907 (3)	0.8877 (3)	0.60874 (12)	0.0673 (12)	
C9	0.3342 (3)	0.8903 (3)	0.56642 (14)	0.0884 (14)	
C10	0.3462 (4)	0.9886 (4)	0.53674 (14)	0.1093 (18)	
C11	0.4152 (5)	1.0848 (4)	0.54957 (16)	0.1011 (18)	
C12	0.4754 (3)	1.0832 (3)	0.59101 (16)	0.0911 (15)	
C13	0.4636 (3)	0.9849 (3)	0.62048 (12)	0.0743 (13)	
C14	0.3725 (6)	1.1963 (5)	0.48060 (16)	0.111 (3)	
C15	0.3360 (3)	0.8142 (3)	0.68660 (11)	0.0880 (15)	
C16	0.1802 (2)	0.6651 (2)	0.71478 (12)	0.0803 (13)	
C17	0.3200 (3)	0.7682 (2)	0.76629 (10)	0.0716 (11)	
H1	0.2912	0.7452	0.6269	0.096*	
H3	0.3322	0.5632	0.6076	0.122*	
H5	0.6741	0.4170	0.6133	0.139*	
H6	0.7401	0.5859	0.6434	0.136*	
H9	0.2867	0.8243	0.5576	0.106*	
H10	0.3074	0.9886	0.5083	0.131*	
H12	0.5244	1.1487	0.5992	0.109*	
H13	0.5048	0.9841	0.6484	0.089*	
H41	0.4606	0.3919	0.6274	0.129*	
H42	0.4839	0.4334	0.5758	0.129*	
H71	0.5880	0.7057	0.6771	0.116*	
H72	0.6147	0.7538	0.6266	0.116*	
H101	0.3533	0.6564	0.7163	0.067*	
H141	0.4108	1.1424	0.4589	0.133*	
H142	0.3766	1.2789	0.4694	0.133*	
H143	0.2899	1.1728	0.4843	0.133*	
H151	0.2683	0.8699	0.6847	0.106*	
H152	0.4049	0.8569	0.6993	0.106*	
H161	0.1657	0.6066	0.7389	0.096*	
H162	0.1726	0.6256	0.6853	0.096*	
H163	0.1227	0.7303	0.7170	0.096*	
H171	0.3986	0.8040	0.7684	0.086*	
H172	0.3122	0.7047	0.7890	0.086*	
H173	0.2603	0.8299	0.7717	0.086*	

### Atomic displacement parameters (Å^2^)

**Table:**

	*U*^11^	*U*^22^	*U*^33^	*U*^12^	*U*^13^	*U*^23^
Cl1	0.0623 (5)	0.0523 (4)	0.1064 (6)	0.0042 (4)	−0.0143 (5)	0.0124 (5)
O1	0.131 (4)	0.092 (2)	0.103 (2)	0.038 (2)	0.040 (2)	0.0221 (18)
N1	0.0502 (18)	0.0466 (15)	0.0711 (17)	0.0117 (13)	0.0113 (14)	0.0100 (14)
C1	0.104 (4)	0.053 (2)	0.082 (2)	0.022 (2)	0.022 (2)	0.011 (2)
C2	0.077 (2)	0.054 (2)	0.063 (2)	0.012 (2)	0.007 (2)	0.0001 (17)
C3	0.086 (3)	0.061 (2)	0.158 (3)	0.008 (2)	−0.018 (2)	−0.036 (2)
C4	0.123 (5)	0.065 (3)	0.134 (5)	0.000 (3)	0.001 (4)	−0.009 (3)
C5	0.116 (5)	0.102 (6)	0.129 (8)	0.013 (4)	−0.003 (5)	−0.003 (5)
C6	0.099 (4)	0.108 (5)	0.134 (7)	0.011 (4)	−0.005 (4)	−0.009 (5)
C7	0.101 (3)	0.104 (3)	0.085 (2)	−0.004 (2)	−0.017 (2)	−0.013 (2)
C8	0.089 (2)	0.050 (2)	0.063 (2)	0.017 (2)	0.020 (2)	0.0053 (18)
C9	0.112 (3)	0.082 (2)	0.071 (2)	0.000 (2)	0.005 (2)	−0.012 (2)
C10	0.155 (4)	0.111 (3)	0.062 (2)	0.047 (3)	0.008 (2)	0.004 (2)
C11	0.162 (5)	0.063 (2)	0.078 (3)	0.036 (3)	0.050 (3)	0.020 (2)
C12	0.125 (3)	0.061 (2)	0.088 (2)	−0.005 (2)	0.031 (2)	−0.007 (2)
C13	0.099 (3)	0.058 (2)	0.066 (2)	0.010 (2)	0.015 (2)	0.002 (2)
C14	0.133 (9)	0.105 (6)	0.095 (3)	0.036 (6)	0.035 (4)	0.036 (3)
C15	0.109 (3)	0.071 (2)	0.084 (2)	0.026 (2)	0.032 (2)	0.029 (2)
C16	0.050 (2)	0.067 (2)	0.124 (3)	−0.004 (2)	−0.016 (2)	−0.010 (2)
C17	0.068 (2)	0.069 (2)	0.077 (2)	0.000 (2)	−0.0014 (18)	−0.0100 (18)

### Geometric parameters (Å, °)

**Table:**

O1—C11	1.374 (5)	C3—H3	0.930
O1—C14	1.408 (6)	C4—H41	0.970
N1—C15	1.488 (4)	C4—H42	0.970
N1—C16	1.479 (4)	C5—H5	0.930
N1—C17	1.481 (3)	C6—H6	0.930
C1—C2	1.511 (5)	C7—H71	0.970
C1—C8	1.513 (4)	C7—H72	0.970
C1—C15	1.478 (4)	C9—H9	0.930
C2—C3	1.315 (5)	C10—H10	0.930
C2—C7	1.492 (4)	C12—H12	0.930
C3—C4	1.496 (6)	C13—H13	0.930
C4—C5	1.493 (8)	C14—H141	0.960
C5—C6	1.286 (8)	C14—H142	0.960
C6—C7	1.451 (6)	C14—H143	0.960
C8—C9	1.378 (5)	C15—H151	0.970
C8—C13	1.378 (5)	C15—H152	0.970
C9—C10	1.381 (6)	C16—H161	0.960
C10—C11	1.354 (7)	C16—H162	0.960
C11—C12	1.375 (6)	C16—H163	0.960
C12—C13	1.379 (5)	C17—H171	0.960
N1—H101	0.860	C17—H172	0.960
C1—H1	0.980	C17—H173	0.960
			
C11—O1—C14	118.2 (3)	C6—C5—H5	118.3
C15—N1—C16	115.9 (2)	C5—C6—H6	118.4
C15—N1—C17	105.9 (2)	C7—C6—H6	118.4
C16—N1—C17	110.5 (2)	C2—C7—H71	107.8
C2—C1—C8	115.2 (3)	C2—C7—H72	107.8
C2—C1—C15	118.2 (2)	C6—C7—H71	107.8
C8—C1—C15	111.4 (2)	C6—C7—H72	107.8
C1—C2—C3	119.3 (3)	H71—C7—H72	109.5
C1—C2—C7	120.3 (3)	C8—C9—H9	119.1
C3—C2—C7	120.4 (3)	C10—C9—H9	119.1
C2—C3—C4	123.8 (4)	C9—C10—H10	120.4
C3—C4—C5	113.0 (4)	C11—C10—H10	120.4
C4—C5—C6	123.3 (5)	C11—C12—H12	119.9
C5—C6—C7	123.3 (5)	C13—C12—H12	119.9
C2—C7—C6	116.1 (3)	C8—C13—H13	119.9
C1—C8—C9	116.9 (3)	C12—C13—H13	119.9
C1—C8—C13	124.9 (3)	O1—C14—H141	109.5
C9—C8—C13	118.1 (3)	O1—C14—H142	109.5
C8—C9—C10	121.7 (3)	O1—C14—H143	109.5
C9—C10—C11	119.2 (4)	H141—C14—H142	109.5
O1—C11—C10	124.4 (4)	H141—C14—H143	109.5
O1—C11—C12	115.2 (4)	H142—C14—H143	109.5
C10—C11—C12	120.4 (4)	N1—C15—H151	107.4
C11—C12—C13	120.3 (3)	N1—C15—H152	107.4
C8—C13—C12	120.3 (3)	C1—C15—H151	107.4
N1—C15—C1	117.4 (2)	C1—C15—H152	107.4
C15—N1—H101	108.1	H151—C15—H152	109.5
C16—N1—H101	108.1	N1—C16—H161	109.5
C17—N1—H101	108.1	N1—C16—H162	109.5
C2—C1—H1	103.2	N1—C16—H163	109.5
C8—C1—H1	103.2	H161—C16—H162	109.5
C15—C1—H1	103.2	H161—C16—H163	109.5
C2—C3—H3	118.1	H162—C16—H163	109.5
C4—C3—H3	118.1	N1—C17—H171	109.5
C3—C4—H41	108.6	N1—C17—H172	109.5
C3—C4—H42	108.6	N1—C17—H173	109.5
C5—C4—H41	108.6	H171—C17—H172	109.5
C5—C4—H42	108.6	H171—C17—H173	109.5
H41—C4—H42	109.5	H172—C17—H173	109.5
C4—C5—H5	118.3		
			
C14—O1—C11—C10	0.3 (6)	C3—C2—C7—C6	0.2 (3)
C14—O1—C11—C12	180.0 (4)	C7—C2—C3—C4	1.6 (5)
C16—N1—C15—C1	−77.4 (4)	C2—C3—C4—C5	−3.6 (6)
C17—N1—C15—C1	159.8 (3)	C3—C4—C5—C6	4.3 (8)
C2—C1—C8—C9	90.1 (4)	C4—C5—C6—C7	−2.9 (10)
C2—C1—C8—C13	−90.2 (4)	C5—C6—C7—C2	0.5 (8)
C8—C1—C2—C3	−113.9 (3)	C1—C8—C9—C10	177.8 (3)
C8—C1—C2—C7	65.0 (4)	C1—C8—C13—C12	−177.6 (3)
C2—C1—C15—N1	−46.9 (5)	C9—C8—C13—C12	2.1 (5)
C15—C1—C2—C3	110.9 (4)	C13—C8—C9—C10	−1.9 (6)
C15—C1—C2—C7	−70.2 (4)	C8—C9—C10—C11	−0.1 (5)
C8—C1—C15—N1	176.3 (3)	C9—C10—C11—O1	−178.4 (4)
C15—C1—C8—C9	−131.7 (3)	C9—C10—C11—C12	1.9 (7)
C15—C1—C8—C13	48.0 (5)	O1—C11—C12—C13	178.6 (4)
C1—C2—C3—C4	−179.5 (3)	C10—C11—C12—C13	−1.7 (7)
C1—C2—C7—C6	−178.7 (3)	C11—C12—C13—C8	−0.3 (6)

### Hydrogen-bond geometry (Å, °)

**Table:**

*D*—H···*A*	*D*—H	H···*A*	*D*···*A*	*D*—H···*A*
N1—H101···Cl1	0.86	2.28	3.033 (2)	146
C17—H173···Cl1^i^	0.96	2.75	3.641 (3)	155

Symmetry codes: (i) −*x*+1/2, *y*+1/2, *z*.
